# Food for Thought: Evaluating Dietary Documentation in Psychiatric Settings

**DOI:** 10.1192/bjo.2024.593

**Published:** 2024-08-01

**Authors:** Praveen Kumar, Apryl Northrup, Lorna Carroll, Heather Ireland

**Affiliations:** New Craig's Psychiatric Hospital, Inverness, United Kingdom

## Abstract

**Aims:**

This study aims to evaluate dietary history documentation by junior doctors in a psychiatric hospital setting in Scotland. With emerging evidence in nutrition psychiatry highlighting diet's impact on mental health, especially ultra-processed foods, this aspect often receives insufficient attention in clinical assessments. The audit benchmarks current documentation against UK Public Health nutritional guidelines and UK Parenteral & Enteral Nutrition Guidelines on Malnutrition, assessing adequacy and consistency across psychiatric diagnoses.

**Methods:**

This audit conducted a systematic review of medical records in psychiatric wards, focusing on patients newly admitted over six months. The data collection examined admission sheets by junior doctors, covering patient identifiers, admission time, diagnosis, doctor's grade, and comprehensive details on dietary habits, eating behaviours, BMI, and substance use. The review incorporated a dietitian's input to align dietary assessments with UK Public Health Nutritional expectations and the prevention of Malnutrition Guidelines. The goal was to assess the regularity, quantity, variety, and documented changes in patients' dietary behaviours, screening for potential nutrient deficits, impacts of psychotropic medications, and eating disorder psychopathology.

**Results:**

The results showed significant deficiency in the detail and consistency of dietary history documentation across all wards, regardless of the doctors' grade or the patients' psychiatric diagnoses. Most entries were inadequately documented or entirely missing. A particular discrepancy was noted in documenting dietary habits in patients with low BMI or those on metabolic altering antipsychotics, which should necessitate health behavior change dietary interventions. Furthermore, even in severe psychiatric conditions, there was a gap in dietary documentation indicating a widespread oversight in recognising the potential relevance of nutrition in the overall health and treatment planning of psychiatric patients, regardless of the severity or type of their condition.

**Conclusion:**

The audit reveals a gap in psychiatric patient care concerning detailed dietary relevance history documentation. While Scotland's wards routinely use the Malnutrition Universal Screening Tool (MUST) for identifying malnutrition, this tool often overlooks key dietary elements like variety, quantity, and regularity, which are vital for linking diet content to mental health. This oversight is significant given the burgeoning field of nutritional psychiatry. Our findings suggest the necessity for systemic changes to improve dietary history documentation in psychiatric settings. This includes a more structured and systematic approach, integrating insights into the harmful effects of ultra-processed foods on mental health, to provide holistic care.

